# Rethinking electric vehicles in research and planning

**DOI:** 10.1038/s44333-025-00067-z

**Published:** 2025-12-16

**Authors:** Milad Mehdizadeh

**Affiliations:** Institute for Transport Studies, University of Leeds, Leeds, UK

**Keywords:** Climate sciences, Environmental social sciences, Social sciences

## Abstract

Electric vehicles (EVs) are often hailed as climate saviours by some and dismissed as greenwashing by others. This polarization reduces a complex issue to slogans, when what is needed is an evidence-based perspective. EVs matter, but only if researchers and policymakers place them within broader strategies that also address transport planning, equity, and energy systems.

## The polarized electric vehicle (EV) debate

EVs have initiated major debates in both academic and policy circles. Some see them as the key to reducing carbon emissions, cleaning up city air, and cutting our reliance on fossil fuels^1,2^. Others argue that EVs are not as environmentally friendly as they seem^3,4^. They point to the pollution caused by mining materials for batteries, the electricity they consume, the challenge of recycling old batteries, and potential negative spillover effects on sustainability. The debate around EVs often feels polarized, one side claiming EVs will solve all problems and the other insisting they are no better than fossil-fuel cars. Yet this framing hides an important distinction. Not all opposition to EVs is misinformation, but not all criticisms are legitimate either. Clarifying the difference is essential if EVs are to contribute meaningfully to sustainability.

## Misinformation vs. legitimate challenges

Public debates about EVs often mix genuine concerns with misleading claims. Legitimate challenges include well-documented issues such as battery issues (e.g., recycling challenges, environmental impacts of production)^5^, unequal charging access^6^, or the fact that EVs do not solve traffic congestion and car dependency issues^7^. These are real challenges for engineers, policymakers, and planners to address. By contrast, several widely circulated claims are false or exaggerated. Examples include the belief that EVs generate more lifetime emissions than fossil-fuel cars, that they are inherently prone to catching fire, or that batteries are designed to fail early^8^. As an example, studies across multiple countries have shown that such misinformation is widespread, and that conspiracy thinking and mistrust of institutions, rather than scientific illiteracy, are the strongest predictors of whether people accept misinformation about EVs^8^. This distinction can matter because mixing misinformation with legitimate challenges progress in two directions: misinformation weakens public support for electrification, while dismissing legitimate challenges risks repeating past mistakes by promoting a technology without addressing its limitations^9,10^.

## The role of researchers in (re)shaping the debate

What role should researchers play in such debates?^11^ When discussions are polarized, scientists risk being seen either as defenders of the automobile industry or as opponents of electrification. Neither role serves the public well. Our responsibility is to keep the conversation grounded in evidence, highlighting both the benefits and the drawbacks as clearly as possible^12^. That does not mean remaining passive. It means being honest about what we know, cautious about what we do not, and careful not to amplify unnecessary fear^11^. Sometimes the most valuable contribution is to slow things down: to create space for a richer understanding of long-term impacts, rather than rushing to declare a technology either a success or a failure. In the case of EVs, this means recognizing their potential while also taking seriously the social, environmental, and infrastructural challenges they pose. A fact-based stance can help society avoid repeating past mistakes of technological overconfidence while still making the most of real opportunities for sustainable innovation.

This is not just about how we communicate findings, but also about the research questions we choose to ask. With the rise of EVs, hundreds of studies focused on consumer acceptance of adoption in different countries, often without asking whether the energy systems in those regions can realistically sustain large-scale electrification (e.g., see Liao et al.^13^). In many cases, charging EVs may rely heavily on fossil-fuel electricity, resulting in persistently high emissions rather than meaningful reductions, unless the electricity grid is decarbonized in parallel. Another structural challenge is the availability of critical minerals needed for EV production. Recent studies show that supply bottlenecks could limit electrification goals and lead to missed climate targets^14,15^. At the same time, much of such work has overlooked a third structural issue: simply replacing fossil-fuel cars with EVs risks weakening decades of gradual progress to reduce car dependency in cities. Many planners have been working slowly to shift investment toward public transport, cycling, and walkable urban design^16^. A narrative that promotes EVs as the default replacement vehicle can (un)intentionally roll back those efforts, locking societies into car-oriented systems for another generation. To reduce polarization among scientists and ensure more balanced research, we need greater integration and collaboration across disciplines. Projects and funding should actively involve researchers from engineering, economics, social science, and climate studies. Such partnerships can help share concerns, increase awareness of different perspectives, and reduce biased thinking in both research and policy directions.

## Hidden problems with EV growth

Even though EVs are a step forward, they come with (legitimate) challenges that cannot be ignored. Switching to EVs alone does not fix bigger problems like traffic congestion and car dependency. In some cases, it may even make things worse if governments do not invest in public transport, cycling lanes, and pedestrian-friendly cities^16^. Sometimes, EV incentives take funding away from these essential services, making people even more dependent on cars. Some studies have warned about negative spillover effects of EVs on (social) sustainability^7^. EVs might also potentially increase the mobility rates and to some extent substitute for active transport (walking and cycling)^17,18^.

Infrastructure is another issue. Charging stations are expanding, but access is not equal everywhere^6^. Urban centers often have better facilities, while people in rural areas or apartment buildings struggle to charge their vehicles. In addition, a rapid increase in EV adoption, especially in car-dependent countries places significant pressure on the electricity grid. Without careful planning for energy resources, this shift could strain the energy system. While vehicle-to-grid (V2G) technology is often suggested as a solution^19,20^, it remains far from widespread adoption due to barriers like financial disincentives, the need to maintain a minimum guaranteed charge, and concerns over battery degradation^21^. These are not misinformation but real challenges that must be addressed for EV integration to be truly sustainable. If we ignore these effects, we miss the bigger picture. Instead of treating EVs as the only answer, we should focus on a mix of solutions, including public transport, smart city planning, and shorter access to different services/amenities by foot/(e-)bikes in line with X-minute city concepts.

## EVs could be a good option, but not for everyone?

“Segmentation” approaches in transport planning show that the population includes diverse groups of car users/owners^22^. Some segments, such as “complacent car addicts”, enjoy owning and using cars anyway, while others are users/owners primarily motivated by practical utility or necessity^23^. For many in the latter group, especially those living in areas designed around car use, the benefits of ownership outweigh the costs. Another recent study also reports that simply a good accessibility perception by cars to different out-of-home activities/destinations can enhance life satisfaction^24^. Segmentation also applies to preferences for different EV types. While policymakers often favor battery EVs (BEVs) for their potential to deliver the deepest emissions cuts, many drivers remain cautious about their limitations. For some, hybrid EVs (HEVs) or plug-in hybrid EVs (PHEVs) may serve as a more acceptable second-best solution^13^. EV life-cycle benefits can also vary depending on usage patterns and regional electricity mixes. Studies show that policies should consider targeting EV incentives toward households and geographic areas where the potential for emissions reduction is highest^25–28^. So, for car lovers and those who genuinely need a vehicle and care about intrinsic values of cars, different EV types (can) offer a good alternative. They provide a cleaner way to drive and work well for long commutes or areas with limited public transport. But promoting EVs as the default for *everyone* is not the best approach. In many cities, investing in reliable buses, trains, bike paths, and pedestrian-friendly streets would do far more for the environment than simply replacing every fossil-fuel car with an electric one.

Instead of assuming that EVs should be the future for all, we should make them an option (preferably EV *access* than *ownership*) for those who (really) need them while also working to reduce overall car dependency. Here, EV access refers to shared EV options, such as e-carsharing or ridesharing with EVs. This approach allows electrification to meet mobility needs without reinforcing car-centric urban development (e.g., the persistent demand for parking spaces). In this approach, people who do not *really* need vehicles should still have access to EVs through shared services, while the culture of private ownership can be moderated. Determining who *really* needs a vehicle is complex, but national travel surveys can provide, or be adapted to provide, insights into households that rely on cars due to limited alternatives. Policies, both push and pull measures, can then be applied regionally, based on the availability of sustainable transport options, and implemented gradually. A realistic transition requires multiple complementary solutions that consider the needs of all groups and regions. This is in line with new narratives, such as those stated by Anable^29^, who argues that “we must now figure out how to leave little, or ideally no, room for C’AN’T (Car ‘All or Nothing’ Thinking) and replace this with a CAN DO (Cars Are Necessary but Don’t Own) attitude”. The pervasiveness of C’AN’T versus CAN DO thinking can be assessed at both individual and institutional levels. Individual surveys on car dependency or willingness to use shared EVs provide useful indicators, while policy frameworks, funding priorities, and incentive schemes reveal whether ownership remains the dominant assumption. Moreover, for some consumers, purchasing an EV may serve as a form of moral licensing^30^, leading them to feel that they have made a meaningful contribution to environmental protection. This perception might, in turn, justify higher car use or even produce negative spillover effects in other domains, such as increased air travel^31^.

Looking ahead, inspired from recent studies^17,32–34^, I believe that push and redistribution policies should also apply to privately owned EVs in addition to non-EVs. For instance, while congestion charging has primarily targeted non-EVs, extending such measures to EVs could discourage unnecessary EV use in cities, while mechanisms that link EV ownership to contributions toward public transport (e.g., requiring EV owners to subsidize public transport passes for non-car owners) could help balance benefits more fairly. Such measures can support transport equity by ensuring that disadvantaged groups (e.g., lower-income households, car-free households) do not perceive electrification policies as skewed in favor of wealthier households. Perceived inequity can challenge support for climate initiatives, deepen social polarization and weaken trust in institutions, particularly among disadvantaged groups^33,35^. However, these policies might also reduce incentives for EV adoption, face political resistance, and require careful design to avoid unintended consequences^35^. As Anable and Goodwin^35^ highlight, assessing such policies requires a net overall distributive effect approach, which considers not just who pays, but who ultimately benefits.

## (Evidence-based) moderation, not polarization

EVs are part of the path to cleaner transport, but they are not the solution on their own. Treating them as a cure-all risks ignoring limits in energy systems, decline progress on reducing car dependency, and widening inequalities. The real challenge is to place EVs within broader strategies that prioritize public transport, active travel, and sustainable urban design. A thoughtful approach will help ensure that EVs do not just replace one set of problems with another but truly contribute to a more sustainable future.

## Data Availability

No data were used in this study.
